# The Efficacy of Intravitreal Bevacizumab in Vitreous Hemorrhage of Diabetic Subjects

**DOI:** 10.4274/tjo.82542

**Published:** 2016-10-17

**Authors:** Cengiz Alagöz, Yusuf Yıldırım, Murat Kocamaz, Ökkeş Baz, Uğur Çiçek, Burcu Çelik, Halil İbrahim Demirkale, Ahmet Taylan Yazıcı, Muhittin Taşkapılı

**Affiliations:** 1 Beyoğlu Eye Training and Research Hospital, İstanbul, Turkey

**Keywords:** Proliferative diabetic retinopathy, vitreous hemorrhage, intravitreal bevacizumab

## Abstract

**Objectives:**

To evaluate the efficacy of intravitreal bevacizumab (IVB) in the resolution of vitreous hemorrhage (VH) secondary to proliferative diabetic retinopathy (PDR).

**Materials and Methods:**

Seventy eyes of 70 patients (43 male, mean age 55.6±12.2 years) diagnosed with VH secondary to PDR were evaluated retrospectively. Demographic characteristics of the patients, baseline and final clinical results, and the interventions the patients were subject to were recorded. The patients who received IVB injections (group 1, n=29) were compared to those who did not receive injections (group 2, n=41) in terms of VH clearance time and surgery rates.

**Results:**

The mean follow-up time was 14.5±6.1 months in group 1 and 18.4±9.6 months in group 2 (p=0.185). The mean visual acuity was similar between the groups at baseline and at the last visit (for all p>0.05). Panretinal photocoagulation could be applied in 86% of subjects in group 1 and in 58% in group 2 2 within the first month (p=0.016). VH clearance time was not different between the groups (2.3±2.1 months in group 1 and 3.4±2.6 months in group 2, p=0.146). The number of subjects requiring surgery was 7 (24%) in group 1 and 20 (48.8%) in group 2 (p=0.048).

**Conclusion:**

IVB was found effective in cases with VH secondary to PDR in terms of reducing the need for surgery and increasing the rate of subjects to whom panretinal photocoagulation could be applied in the early period, although there was no impact on final visual acuity.

## INTRODUCTION

Diabetic retinopathy (DR) is among the foremost causes of vision loss among working-age adults in developed countries.^1^ The most common causes of vision loss in DR patients are macular edema, vitreous hemorrhage (VH) and tractional retinal detachment (TRD). In DR, angiogenic mediators such as insulin-like growth factor-1, erythropoietin, fibroblast growth factor and vascular endothelial growth factor (VEGF) are released secondary to retinal ischemia and lead to the formation of neovascular structures in the retina.^2,3,4^ VH which arises due to these neovascular structures is an important clinical condition that prevents panretinal photocoagulation (PRP), the gold standard in proliferative (PDR) treatment. Half of PDR patients who do not receive timely treatment develop serious vision loss within 5 years.^5^

Bevacizumab is a humanized monoclonal recombinant anti-VEGF antibody that inhibits all isoforms of VEGF. VEGF has been reported at three times normal levels in the vitreous of advanced PDR patients and is believed to play a key role in neovascularization.^2,6^ Intraocular injection of anti-VEGF drugs induces a rapid regression of retinal and iris neovascularization.^7,8,9,10^ Although currently bevacizumab is usually used to treat macular edema in DR patients, it is also widely used to induce the regression of neovascularization in neovascular glaucoma and before pars plana vitrectomy (PPV).^11,12^ Considering that anti-VEGF therapy accelerates the resolution of hemorrhage and facilitates PRP, it may also be a good choice for patients with VH.

The aim of this study was to evaluate the efficacy of intravitreal bevacizumab (IVB) therapy in patients with PDR-associated VH.

## MATERIALS AND METHODS

The medical records of consecutive patients who were diagnosed with VH due to PDR and showed no signs of TRD on ultrasonography in the retina clinic of our hospital between January 2011 and June 2012 were evaluated retrospectively. In accordance with the Declaration of Helsinki, all patients were informed about the surgical procedures and postoperative period, and written informed consent forms were obtained from all participants.

Vitrectomized eyes, patients with simultaneous bilateral VH, monocular patients, and patients with advanced glaucoma, rubeosis iridis or TRD were excluded from the study. We also excluded patients with any corneal or anterior segment pathology that would affect final visual acuity (VA) or interfere with fundus imaging. IVB was not administered to patients with uncontrolled systemic hypertension, a history of thromboembolism, or a known coagulation disorder, nor to patients with active ocular infection. Patients followed for at least 6 months were included in the study.

Patients’ demographic data (age, gender, ocular and systemic diseases), initial corrected VA and lens status were recorded from their medical records. In addition, we also noted patients’ VA during follow-up, intraocular pressure (IOP) measurements, whether or not PRP could be done, VH status, surgical interventions (injections and PPV), follow-up time and any systemic or local complications. VA was measured using Snellen’s chart and converted to logMAR for statistical analysis. Goldmann applanation tonometer was used for all IOP measurements. Fundus examination was done using a +90 diopter lens.

VH clearance time was defined as the time until primary vessels in the posterior pole and the optic disc were clearly visible and at least 3 quadrants of the peripheral retina were clear enough to perform PRP. Recurrent hemorrhage was defined as hemorrhage developing after the complete resolution of previous hemorrhage and clearance of the fundus.

In our retina clinic, repeated IVB injections are applied to patients when initial IVB treatment does not result in VH clearance allowing PRP early in follow-up, or when VH recurs. Patients are followed for 4-6 months for spontaneous resolution except in cases with bilateral VH, monocular patients, and cases accompanied with rubeosis iridis or TRD. Surgery is indicated in eyes with hemorrhage that has not cleared by the end of this period. VH patients have monthly follow-up examinations and PRP is applied as soon as the fundus clears. When needed, PRP is repeated at subsequent visits. At all follow-up visits, patients without fundus clearing are evaluated for TRD by B-mode ultrasonography. Surgery is performed immediately in patients who develop TRD during follow-up.

### Injection Technique

IVB (1.25 mg/0.05 mL, Altuzan^®^, Roche) injections were done in sterile conditions under topical anesthesia, into the vitreous in the superior temporal quadrant 3.5 mm posterior to the limbus. Light perception was checked after injection and paracentesis was performed when necessary. Topical antibiotic therapy (moxifloxacin eye drops, 4 times daily) was applied for 1 week after injection.

### Surgical Technique

Vitrectomy was recommended for eyes that did not show hemorrhage clearance within the first 4-6 months or developed recurrent VH. Standard 23-gauge PPV was performed under local or general anesthesia. A trocar was placed in the inferotemporal quadrant for infusion. Additional trocars for the oculotome and vitrectomy probe were placed in the superotemporal and superonasal quadrants. After clearing the media of hemorrhage by core vitrectomy, the posterior hyaloid was removed using suction if still attached. Hemostasis was maintained by increasing the infusion pressure or applying laser directly to the extramacular neovascularization. After clearing the peripheral vitreous, endolaser was applied to any areas that could not be adequately cleared. Tamponade was not used in cases without any intraoperative complications; 20% sulfur hexafluoride (SF_6_) gas was applied as a tamponade in cases that developed intraoperative hemorrhage and iatrogenic tear. After removing the trocars the scleral entries were assessed for leakage and any leaking incisions were sutured.

### Statistical Analysis

SPSS version 20.0 (SPSS, Chicago, IL, USA) package software was used in the statistical analysis of all data. Numerical variables are expressed as mean ± standard deviation. Categorical variables are expressed as frequency and percentage (%). The Wilcoxon test was used for dependent intergroup comparisons of numerical variables; the Mann Whitney U test was used for independent comparisons of the two groups. Fisher’s Exact test was used for categorical variables. Results with P values less than 0.05 were accepted as statistically significant.

Patients who were treated by IVB injection and those who were followed without IVB therapy were compared in terms of VA change and final VA, VH clearance time, rate of early PRP application and rate of surgical intervention.

## RESULTS

### Demographic Characteristics

Seventy eyes of 70 patients (43 male, 27 female) who met the inclusion criteria were included in the study. Study subjects were divided into two groups, eyes treated with IVB (group 1, n=29) and eyes not treated with IVB (group 2, n=41). Mean age was 56.2±9.6 years in group 1 and 57.4±10.1 in group 2 (p=0.441). The patients’ preoperative characteristics are summarized in Table 1. Mean follow-up time was 14.5±6.1 months in group 1 and 18.4±9.6 months in group 2 (p=0.185).

### Anatomic Results

IVB therapy was administered to 29 eyes (group 1) at time of diagnosis. Thirteen (44.8%) of these eyes had a repeated IVB injection at an average of 4.1±3.1 months.

PRP could be performed within the first month in 25 eyes in group 1 (86.2%) and in 24 eyes in group 2 (58.5%) that showed partial or complete hemorrhage clearance (p=0.016) (Table 2).

VH clearance time was 2.3±2.1 months in group 1 and 3.4±2.6 months in group 2. Although VH clearance time was shorter in group 1, the different was not statistically significant (p=0.146).

Recurrent hemorrhage developed during follow-up in 4 eyes from group 1 and 4 eyes from group 2. Of the eyes with recurrent hemorrhage from group 1, PPV was performed in 3 of the eyes and IVB injection was repeated in the other. Of the group 2 eyes with recurrent hemorrhage, 2 underwent PPV and 2 were followed.

Seven patients (24.1%) from group 1 and 20 (48.8%) from group 2 underwent PPV due to persistent or recurrent VH (p=0.048) (Table 2). The proportion of patients that underwent PPV was significantly higher in group 2.

### Functional Results

Initial VA was 1.83±1.0 logMAR in group 1 and 2.15±0.9 logMAR in group 2 (p=0.08). VA at final examination was 0.78±0.7 logMAR in group 1 and 0.69±0.5 logMAR in group 2 (p=0.925). Difference between initial and final VA was 1.05±1.0 logMAR in group 1 and 1.45±0.9 in group 2 (p=0.118) (Table 3).

### Complications

During follow-up, 2 eyes (6.9%) from group 1 developed epiretinal membrane (ERM) and 4 (13.7%) developed cataract; in group 2, ERM formed in 1 eye (2.4%) and cataract in 3 eyes (7.3%) (p=0.299). None of the patients developed neovascular glaucoma due to iris or angle neovascularization. No systemic complications associated with IVB injection were observed during follow-up.

## DISCUSSION

**Figure 1:**
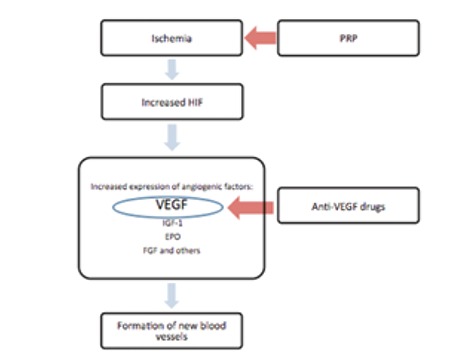
Pathophysiology of proliferative diabetic retinopathy HIF: Hypoxia-inducible factor, VEGF: Vascular endothelial growth factor, IGF-1: Insulin-like growth factor-1, EPO: Erythropoietin, FGF: Fibroblast growth factor, PRP: Panretinal photocoagulation

Retinal ischemia underlies the pathophysiology of PDR. Hypoxia-inducible factor released secondary to retinal hypoxia and ischemia increases expression of angiogenic factors such as VEGF, insulin-like growth factor-1 and erythropoietin,^2,3,4^ thereby leading to the formation of new vessels and fibrovascular structures (Figure 1). Contraction of the fibrous component can increase the tendency of neovascular structures to bleed and, in advanced cases, lead to TRD. PRP is currently accepted as the gold standard in PDR treatment.^13,14^ However, photocoagulation therapy may not be possible in eyes with intravitreal opacity such as VH. This condition leads to the progression of neovascular tissue and may result in persistent VH, recurrent VH, or development of TRD. Currently, in patients whose hemorrhage does not clear spontaneously, PRP is performing after clearing the media by vitrectomy.

In recent years, intravitreal anti-VEGF agents have been used in DR treatment for diabetic macular edema, preretinal hemorrhage, active neovascularization and as preoperative adjuvant therapy in PDR cases.^9,10,11,12,15,16,17^ Anti-VEGF drugs prevent the formation of new vasculature by directly affecting VEGF, but reducing retinal ischemia is not among their functions (Figure 1). It has been reported that the efficacy of anti-VEGF agents is transitory when used alone to treat PDR, and effective long-term results were only attainable when these drugs are used in addition to PRP.^18^

With the present study, we aimed to evaluate the effects of IVB on hemorrhage clearance time, need for surgery and final VA results in diabetic patients with VH. Our results indicate that IVB therapy does not significantly impact clearance time (2.3 months in group 1 and 3.4 months in group 2, p=0.146), but decreases the number of cases requiring surgery (24% in group 1 and 49% in group 2, p=0.048). Reports in the literature regarding the effect of anti-VEGF drugs in VH are equivocal.^19,20,21^ Huang et al.^19^ found that bevacizumab therapy reduced both clearance time and the surgery rate in VH patients. In a study by the Diabetic Retinopathy Clinical Research Network (DRCRnet), researchers applied intravitreal ranibizumab to 125 eyes and intravitreal saline to 136 eyes of diabetic patients with VH that precluded PRP. At 16 weeks after injection, eyes injected with ranibizumab showed a greater improvement in VA, lower rate of recurrent hemorrhage and significantly higher rate of PRP completion compared to the saline-injected group.^20^ However, in a later report of results at 1 year, they reported no significant differences in VA outcomes or surgery rates.^21^

Intravitreal anti-VEGF drugs are known to have a half-life of 7-10 days in the eye^22,23^ and their clinical efficacy is as short as 4 weeks.^23,24^ As anti-VEGF agents block new vessel formation and also induce regression of existing vessels,^9,10,25^ they can theoretically prevent new hemorrhages from preexisting or new loci in VH patients. Thus, injection of anti-VEGF drugs facilitates clearing of the media and should allow the application of PRP in more patients in the early phase. Consistent with the results reported in the DRCRnet study, in the present study we observed a higher rate of PRP completion in the first month in patients treated with IVB. Because of the drug’s short half-life, it is expected that IVB is not effective against recurrent hemorrhage in the long term. The completion of laser photocoagulation, the current gold standard therapy for PDR, remains important for the long-term prevention of recurrent hemorrhage.^13,14^ In our study, the rates of recurrent hemorrhage during follow-up were comparable in the two groups (13.8% in group 1 and 9.8% in group 2). However, in group 2 PRP could be completed in a larger proportion of patients after vitrectomy and media clearance.

The ideal interval for repeated IVB injections in cases of PDR-associated VH has not been clearly established. Huang et al.^19^ applied a second injection in eyes not exhibiting signs of hemorrhage clearance 4-6 weeks after the first injection. Although the injection interval could not be standardized due to the retrospective nature of our study, repeated IVB injections were administered to eyes that did not show reduced hemorrhage or developed recurrent hemorrhage in early follow-up. Prospective, controlled studies are needed to determine the efficacy and frequency of repeated injections for the treatment of VH associated with PDR.

In general, the VA of PDR patients can be expected to return to pre-hemorrhage levels after the VH is completely resorbed. Huang et al.^19^ found that eyes injected with IVB and those not injected showed similar improvements in VA in PDR-associated VH patients followed for at least 12 months. DRCRnet reported that VA improved more in the ranibizumab-injected group in the short term, but found no difference between the two groups in the long term.^20,21^ Similarly, in our case series we observed comparable results in VA improvement and final VA levels between the two groups. Our findings that bevacizumab injection does not result in a significant difference in final VA support results obtained by other researchers. It has been demonstrated in previous studies that VA loss secondary to PRP is attenuated when anti-VEGF therapy is applied in combination to PRP in diabetic patients.^26,27,28^ However, both the present study and previous ones^20,22^ could not demonstrate any positive effect of anti-VEGF drugs on visual function in VH patients.

## CONCLUSION

IVB was found effective in cases with VH secondary to PDR in terms of reducing the need for surgery and increasing the rate of PRP completion in the early period, but it did not impact final VA.

### Ethics

Ethics Committee Approval: Retrospective study, Informed Consent: It was taken.

Peer-review: Externally peer-reviewed.

## Figures and Tables

**Table 1:**
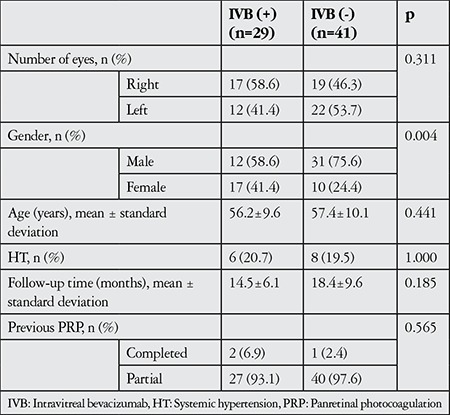
Patients’ demographic characteristics and preoperative findings

**Table 2:**
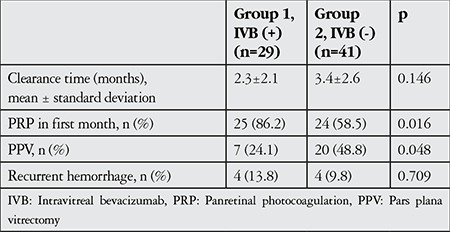
Clinical findings in eyes treated with intravitreal bevacizumab and eyes not treated with intravitreal bevacizumab

**Table 3:**
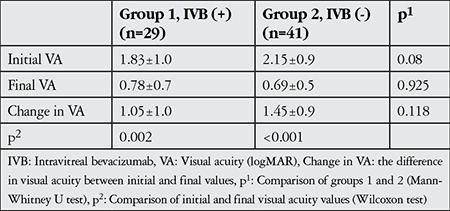
Visual acuity changes in the study groups

## References

[1] Klein R (1992). Retinopathy in a population-based study. Trans Am Ophthalmol Soc..

[2] Aiello LP, Avery RL, Arrigg PG, Keyt BA, Jampel HD, Shah ST, Pasquale LR, Thieme H, Iwamoto MA, Park JE, et al (1994). Vascular endothelial growth factor in ocular fluid of patients with diabetic retinopathy and other retinal disorders. N Engl J Med..

[3] Sivalingam A, Kenney J, Brown GC, Benson WE, Donoso L (1990). Basic fibroblast growth factor levels in the vitreous of patients with proliferative diabetic retinopathy. Arch Ophthalmol..

[4] Meyer-Schwickerath R, Pfeiffer A, Blum WF, Freyberger H, Klein M, Losche C, Rollmann R, Schatz H (1993). Vitreous levels of the insulin-like growth factors I and II, and the insulin-like growth factor binding proteins 2 and 3, increase in neovascular eye disease. Studies in nondiabetic and diabetic subjects. J Clin Invest..

[5] Chew EY, Regillo CD, Brown GC, Flyn HW (1999). Major clinical trials of vitreoretinal diseases. Vitreoretinal Disease: The Essentials.

[6] Malik RA, Li C, Aziz W, Olson JA, Vohra A, McHardy KC, Forrester JV, Boulton AJ, Wilson PB, Liu D, McLeod D, Kumar S (2005). Elevated plasma CD105 and vitreous VEGF levels in diabetic retinopathy. J Cell Mol Med..

[7] Avery RL, Pearlman J, Pieramici DJ, Rabena MD, Castellarin AA, Nasir MA, Giust MJ, Wendel R, Patel A (2006). Intravitreal bevacizumab (Avastin) in the treatment of proliferative diabetic retinopathy. Ophthalmology..

[8] Jorge R, Costa RA, Calucci D, Cintra LP, Scott IU (2006). Intravitreal bevacizumab (Avastin) for persistent new vessels in diabetic retinopathy (IBEPE study). Retina..

[9] Avery RL (2006). Regression of retinal and iris neovascularization after intravitreal bevacizumab (Avastin) treatment. Retina..

[10] Isaacs TW, Barry C (2006). Rapid resolution of severe disc new vessels in proliferative diabetic retinopathy following a single intravitreal injection of bevacizumab (Avastin). Clin Exp Ophthalmol..

[11] Haritoglou C, Kook D, Neubauer A, Wolf A, Priglinger S, Strauss R, Gandorfer A, Ulbig M, Kampik A (2006). Intravitreal bevacizumab (Avastin) therapy for persistent diffuse diabetic macular edema. Retina..

[12] Ishikawa K, Honda S, Tsukahara Y, Negi A (2009). Preferable use of intravitreal bevacizumab as a pretreatment of vitrectomy for severe proliferative diabetic retinopathy. Eye (Lond)..

[13] Early photocoagulation for diabetic retinopathy (1991). ETDRS report number 9. Early Treatment Diabetic Retinopathy Study Research Group. Ophthalmology..

[14] Kapran Z, Acar N (2008). Proliferatif Diyabetik Retinopati Tedavisi. Ret-Vit..

[15] Shih CW, Yang CM, Chen MS, Wang TJ (2008). Intravitreal injection of bevacizumab and gas for diabetic premacular hemorrhage with active fibrovascular proliferation. Graefes Arch Clin Exp Ophthalmol..

[16] Alakuş MF, Taş M ÖV, İşcan Y, Türkçü FM, Şimşek A, Ünlü MK (2012). Diabetik Maküla Ödeminde İntravitreal Bevacizumab Etkinliğininin Değerlendirilmesi. Ret-Vit..

[17] Yüksel K, Baz Ö, Çelik U, Herdem U, Alagoz C, Özgürhan EB, Yazıcı AT, Demirok A (2015). Diyabetik traksiyonel retina dekolmanlı olgularda 23-gauge pars plana vitrektomi cerrahisi sonuçları. Journal of Clinical and Experimental Investigations..

[18] Arevalo JF (2013). Intravitreal bevacizumab as anti-vascular endothelial growth factor in the management of complications of proliferative diabetic retinopathy. Med Hypothesis Discov Innov Ophthalmol..

[19] Huang YH, Yeh PT, Chen MS, Yang CH, Yang CM (2009). Intravitreal bevacizumab and panretinal photocoagulation for proliferative diabetic retinopathy associated with vitreous hemorrhage. Retina..

[20] Diabetic Retinopathy Clinical Research N (2013). Randomized clinical trial evaluating intravitreal ranibizumab or saline for vitreous hemorrhage from proliferative diabetic retinopathy. JAMA Ophthalmol..

[21] Bhavsar AR, Torres K, Glassman AR, Jampol LM, Kinyoun JL (2014). Evaluation of results 1 year following short-term use of ranibizumab for vitreous hemorrhage due to proliferative diabetic retinopathy. JAMA Ophthalmol..

[22] Krohne TU, Liu Z, Holz FG, Meyer CH (2012). Intraocular pharmacokinetics of ranibizumab following a single intravitreal injection in humans. Am J Ophthalmol..

[23] Stewart MW (2014). Pharmacokinetics, pharmacodynamics and pre-clinical characteristics of ophthalmic drugs that bind VEGF. Expert Rev Clin Pharmacol..

[24] Miyake T, Sawada O, Kakinoki M, Sawada T, Kawamura H, Ogasawara K, Ohji M (2010). Pharmacokinetics of bevacizumab and its effect on vascular endothelial growth factor after intravitreal injection of bevacizumab in macaque eyes. Invest Ophthalmol Vis Sci..

[25] Arevalo JF, Wu L, Sanchez JG, Maia M, Saravia MJ, Fernandez CF, Evans T (2009). Intravitreal bevacizumab (Avastin) for proliferative diabetic retinopathy: 6-months follow-up. Eye (Lond)..

[26] Filho JA, Messias A, Almeida FP, Ribeiro JA, Costa RA, Scott IU, Jorge R (2011). Panretinal photocoagulation (PRP) versus PRP plus intravitreal ranibizumab for high-risk proliferative diabetic retinopathy. Acta Ophthalmol..

[27] Cho WB, Oh SB, Moon JW, Kim HC (2009). Panretinal photocoagulation combined with intravitreal bevacizumab in high-risk proliferative diabetic retinopathy. Retina..

[28] Mason JO, Yunker JJ, Vail R, McGwin G (2008). Intravitreal bevacizumab (Avastin) prevention of panretinal photocoagulation-induced complications in patients with severe proliferative diabetic retinopathy. Retina..

